# Evaluation of the intraventricular hemodynamics of patients with left ventricular dysfunction via vector flow mapping

**DOI:** 10.3389/fcvm.2025.1617482

**Published:** 2025-10-15

**Authors:** Qun Xu, Li Hao, Guang Yang, Mei Zhang, Mingxue Di

**Affiliations:** ^1^ Department of Gerontology, The First Affiliated Hospital of Shandong First Medical University & Shandong Provincial Qianfoshan Hospital, Jinan, Shandong, China; ^2^ National Key Laboratory for Innovation and Transformation of Luobing Theory, the Key Laboratory of Cardiovascular Remodeling and Function Research, Chinese Ministry of Education, Chinese National Health Commission and Chinese Academy of Medical Sciences, Department of Cardiology, Qilu Hospital of Shandong University, Jinan, China

**Keywords:** vorticity, vector flow mapping, left ventricular dysfunction, hemodynamics, cardiac function

## Abstract

**Background:**

Vorticity, measured via new vector flow mapping (VFM), a quantitative marker of vortex dynamics, can reflect hemodynamic changes more sensitively, potentially offering complementary information to conventional echocardiographic indices of cardiac function.

**Aims:**

We investigated left ventricular hemodynamics in both normal subjects and patients with left ventricular dysfunction to explore the probability of evaluating cardiac function with the assistant index, the highest vorticity value of a vortex (Vort-max).

**Methods:**

Sixty subjects were divided into 3 groups, namely, the control group, Group I (HFpEF) and Group II (HFmrEF&HFrEF), and examined via conventional echocardiography. VFM was performed from the apical 5-chamber view to calculate the vorticity during diastole and systole in the left ventricle.

**Results:**

Hemodynamic changes were obvious during diastolic dysfunction. The Vort-max-base values of early and late diastole in Group I were greater than those in the control groups (*P* < 0.001). The Vort-max-apex of mid-systole in Group I was greater than that in the control group (*P* = 0.044). Vort-max-base in early diastole, Vort-max in all three segments in mid-diastole, Vort-max-apex and Vort-max-middle in late diastole, were associated with *E*/*e*′ and *E* (*P* < 0.05). Vort-max-middle and Vort-max-base in all three segments in late diastole was associated with A (*P* < 0.05).

**Conclusions:**

Blood flow energy was detected in patients with diastolic and systolic dysfunction by using Vort-max derived from vector flow mapping. The vorticity value could be a novel parameter for evaluating the hemodynamic changes in the left ventricular cavity and cardiac diastolic function.

## 1 Introduction

Cardiac hemodynamics can be modified by valves, chamber geometry, and wall motions. Various cardiac diseases lead to impaired cardiac function, which induces abnormal blood flow. Abnormal hemodynamics may aggravate cardiac function and lead to myocardial remodeling. Thus, pathological blood flow may be both a consequence and a cause of cardiac diseases (1).

Previous investigations confirmed that indices derived from conventional color Doppler flow imaging and tissue Doppler imaging had important prognostic value in the evaluation of patients with cardiac dysfunction, although these methods depend on the Doppler angle. To bypass this impediment of angle dependency, several new technologies, such as cardiac computed tomography, magnetic resonance imaging (MRI) and contrast echocardiography using particle image velocimetry (CE-PIV), have been developed and have already been used in clinical diagnosis and treatment in the early stages of cardiac diseases or for assessment after cardiac surgery (2).

The investigation of intraventricular flow and vortex dynamics has been pioneered by 4D Flow MRI, which provides high-resolution, three-dimensional information on left ventricular (LV) vorticity and has yielded important pathophysiological insights (3, 4). While these techniques provide comprehensive data, their application can be limited by factors including longer total procedure times, the need for specialized equipment and personnel, and unsuitability for unstable patients. Vector flow mapping (VFM), in contrast, can be seamlessly integrated into a standard bedside echocardiographic examination, adding only minutes to the acquisition time (5).

On the basis of color Doppler flow imaging and a series of mathematical equations, VFM estimates the intraventricular flow velocity vector without angle dependency and allows instantaneous visualization of flow vectors (6–8). The accuracy of velocity vectors computed by the VFM has been verified by comparing them with values computed from particle image velocimetry (PIV) (7). The mathematical calculations of the VFM are based on two assumptions: that flow along each scan is laminar and through plane flow is negligible and that blood is an incompressible, laminar Newtonian fluid. The VFM obtains velocity component data in the beam direction via color Doppler and perpendicular to the beam via wall-motion speckle tracking (7–9). Hence, the newly developed VFM provides more dynamic and objective information about blood flow, which dynamically and objectively reflects hemodynamic changes in the cardiac chambers during the cardiac cycle.

The vortex is the circular or swirling movement of a whirling fluid around a virtual central axis. The main functions of the vortex are to reduce energy loss and optimize cardiac function. Furthermore, the vortex contributes to atrioventricular coupling and the redirection of blood flow toward outflow tracts, which keeps blood from potential stasis (1, 10). Different indices of LV vortex characteristics have been studied in different clinical phases of the cardiac cycle (11–15), and reports indicate that the vortex can mirror cardiac function to some degree (1, 10, 16–19). The generation and evolution of the vortex in turn affect cardiac function (17, 20).

While established parameters such as the *E*/*e*′ ratio are pivotal, a significant portion of patients fall into an ‘indeterminate’ category, posing a diagnostic challenge (21). This highlights an unmet clinical need for more direct, non-invasive measures of left ventricular (LV) hemodynamics. We therefore positioned our study not as the first to investigate LV vorticity *per se*, but as an evaluation of VFM as a bedside, cost-effective echocardiographic method for quantifying LV vorticity.

We hypothesize that Vort-max, a parameter reflecting the peak rotational energy of these vortices, could serve as a sensitive marker of mechanical inefficiency, potentially aiding in the earlier detection of cardiac dysfunction, refining patient risk stratification, and monitoring therapeutic responses.

Vort-max, derived from VFM within the LV vortex, is a novel flow dynamic parameter that reflects the motion energy of blood flow. A quantitative assessment of blood flow in terms of Vort-max may provide insight into left ventricular function (16). However, the real hemodynamics at different cardiac function levels is unclear. Moreover, the evolution of Vort-max and its relationship with cardiac function have not been quantitatively investigated.

Accordingly, this study uses VFM to investigate LV hemodynamic changes in terms of Vort-max throughout the cardiac cycle in both normal subjects and patients with left ventricular dysfunction and to explore the potential probability of evaluating cardiac function with the assistant index Vort-max.

## 2 Methods

### 2.1 Study population

**Figure 1:**
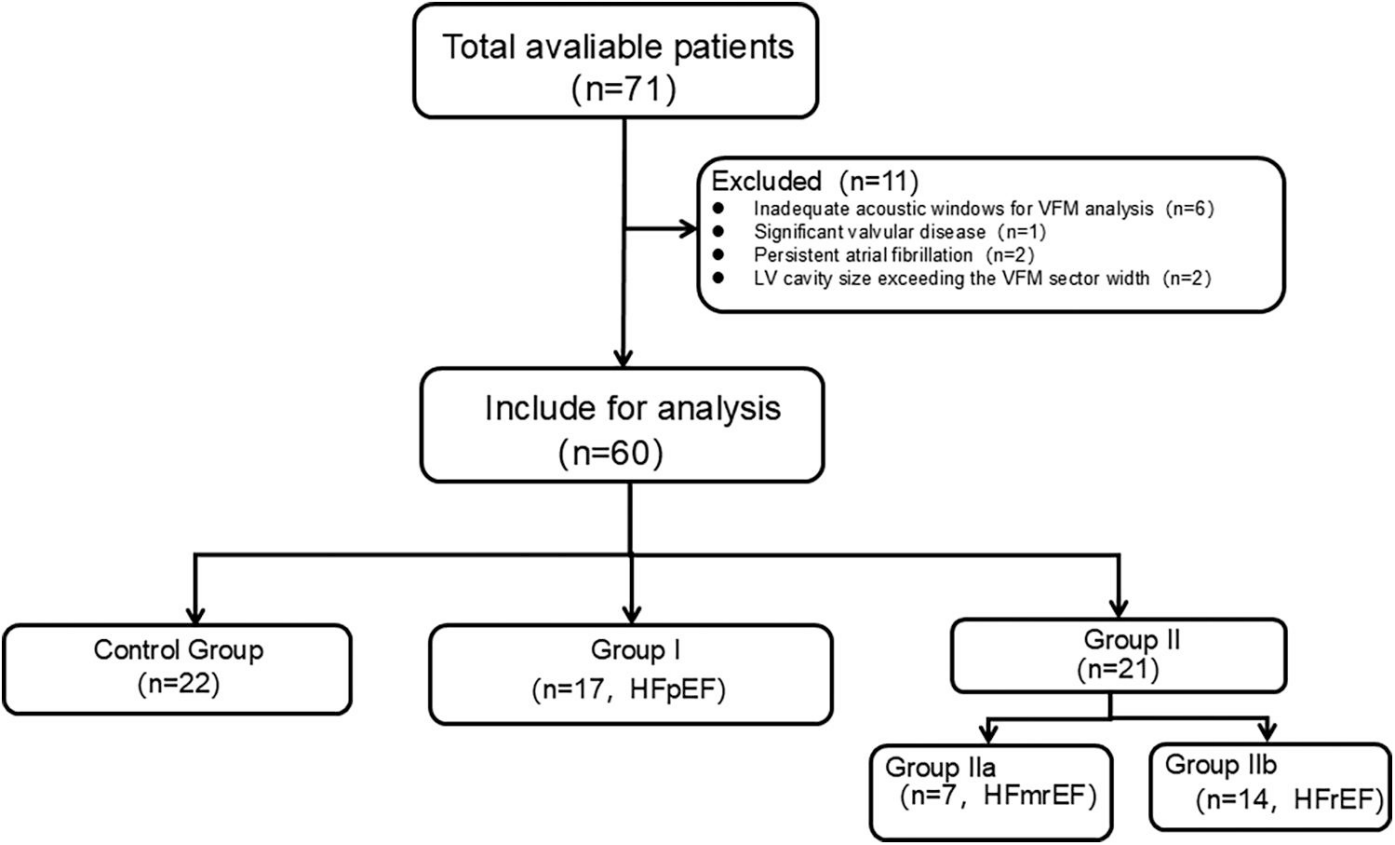
A flow diagram detailing participant enrollment and allocation.

A total of 71 subjects referred for echocardiography were screened for potential inclusion. The exclusion criteria were as follows: (1) insufficient quality echocardiographic images; (2) unstable clinical or hemodynamic status; or (3) nonsinus rhythm, significant aortic regurgitation and stenosis, mitral valvular disease, moderate-to-severe pulmonary hypertension, congenital heart disease, myocarditis, or pericarditis. Of these, 11 subjects were excluded due to the following reasons: inadequate acoustic windows for VFM analysis (*n* = 6), significant (greater than moderate) valvular disease (*n* = 1), persistent atrial fibrillation (*n* = 2), and LV cavity size exceeding the VFM sector width (*n* = 2). The final study cohort comprised 64 subjects who met all inclusion and exclusion criteria. The subjects were divided into 3 groups as recommended in the guidelines for echocardiographic examination in EAE/ASE (21). This cross-sectional study included 22 controls [Control Group], 17 patients [Group I] and 21 patients [Group II]. Patients in Control Group were diagnosed with no history of cardiovascular diseases, no symptoms or signs of cardiac diseases, and normal electrocardiogram and echocardiography results. Patients in Group I all met the criteria for heart failure with preserved ejection fraction (HFpEF; LVEF ≥50%).Patients in Group II the criteria for heart failure with reduced ejection fraction (HFmrEF or HFrEF; LVEF <50%), which were sub-classified into heart failure with mildly reduced ejection fraction (HFmrEF; LVEF 40%–49%) (Group IIa)and heart failure with reduced ejection fraction (HFrEF; LVEF <40%) (Group IIb).All grouping criteria refer to the recommendations of the 2016 ASE/EACVI guidelines (22) and 2025 ASE guidelines (23). Furthermore, diastolic dysfunction for all patients in Group I and Group II was graded as Grade I, II, or III also based on the above recommendations (22, 23). A flow diagram detailing participant enrollment and allocation was shown in Figure 1.

All clinical data, including sex, age, weight, height, and echocardiographic measurements, were collected. Body mass index (BMI) was calculated as weight (kg) divided by height (m^2^). Blood pressure (BP) was measured by an electronic sphygmomanometer after the participants had rested in the supine position for at least 10 min. The study was approved by the local medical ethics committee, and written consent was obtained from all patients.

### 2.2 Echocardiographic examinations

All the subjects underwent conventional transthoracic echocardiography and noninvasive intracardiac flow assessment with VFM in the standard left lateral decubitus position on a ProSound F75 system (Hitachi Aloka Medical Ltd., Tokyo, Japan), which was synchronously connected to an ECG and equipped with a UST-52105 transducer (1–5 MHz). The echocardiography was performed by experienced **sonographers**.

Basic echocardiographic data (left ventricle morphology, conventional systolic parameters and diastolic parameters) were acquired according to the recommendations (24).

The left ventricular end-diastolic diameter (Dd), intraventricular septum wall thickness diameter at end-diastole (IVSTd), and posterior wall thickness diameter at end-diastole (PWTd) were measured in the parasternal long-axis view. The left ventricular mass was calculated according to Devereux’s formula (25), LVMI (g/m^2^) = (1.04 × [(IVST + LVID + PWT)^3^ − LVID^3^] − 13.6 g)/body surface area. The left ventricular end-diastolic volume (EDV), end-systolic volume (ESV) and LV ejection fraction were obtained via biplane Simpson’s rule. Stroke volume (SV) was the difference between the EDV and ESV. The sphericity index was calculated as the ratio of the short- to long-axis dimensions in the four-chamber view at end-diastole. The long-axis dimension was measured from the apex to the middle of the mitral valve annulus, and the short-axis dimension was measured at the point where it perpendicularly intersects the midpoint of the long axis.

In the apical four-chamber view, early filling and late filling peak wave velocities (*E*, *A*) and deceleration time of *E* (EDT) were recorded via pulsed-wave Doppler with the sample volume set at the tips of the mitral leaflet, whereas early transmitral annular lateral velocity (*e*), the index of LV relaxation, was measured via tissue Doppler imaging. The flow propagation velocity (*Vp*) was measured via color Doppler imaging with the M-mode cursor placed through the center of the transmitral flow in the slope of the first aliasing velocity during early filling. The ratios of *E* to *e* (*E*/*e*) and *E*/*Vp* were calculated as indices of the LV filling pressure or LV diastolic stiffness (24). The sphericity index was calculated as the ratio of short- to long-axis dimensions in the four-chamber view at end-diastole (26, 27).

### 2.3 Vector flow mapping

To calculate the intensity and direction of flow, VFM velocity data were derived from Doppler. Color Doppler is only able to detect velocity components parallel to the echo beam. A series of mathematical algorithms was implemented to obtain the velocity component perpendicular to the echo beam. The VFM has been proven to be a reasonable tool for depicting and measuring *in vitro*-generated fluid (7, 8, 28).

The images were recorded in the apical three-chamber view with color Doppler, including the whole LV, mitral, and aortic valves in the scan plane. The Nyquist limit for 2D color Doppler imaging was set high enough to mitigate the aliasing phenomenon as much as possible. Efforts were made to ensure that the scan plane crossed both the mitral and aortic valves to approximate both the gross flow properties and minor secondary flows. To obtain the maximized frame rates in all the subjects (>25 frames/s) and to encompass the entire LV cavity, the width of the ultrasound scan, imaging depth and spatiotemporal characteristics were optimized. Cine loops of 3 consecutive cycles were stored digitally and analyzed offline with DAS-RS1 5.0 software (Hitachi Aloka Medical Ltd.). After initial processing (cavity–endocardial border tracking on the special frame, speckle tracking to automatically determine the cardiac wall motion, and manual aliasing corrections), the region of interest (ROI) was determined by tracing, and Vort-max was automatically detected by the analysis software for each frame. The vorticity (in units of s^−1^) is defined as the curl of velocity *v* (ω=∇×v) and represents the local rotational motion of a fluid at a particular spatial point. This equation shows that vorticity changes at points where the size and direction of the velocity vectors change. Vort-max is a value that indicates the vorticity of a vortex with the highest energy at a particular spatial position. For our analysis, we extracted the maximum absolute vorticity value (Vort-max, in units of s⁻¹) within the dominant intraventricular vortex for each specific cardiac phase. Vort-max therefore represents the peak rotational intensity of the vortex and was automatically calculated by the DAS-RS1 analysis software. A high value of Vort-max indicates that there is rapid velocity or that there is considerable turbulence.

**Figure 2:**
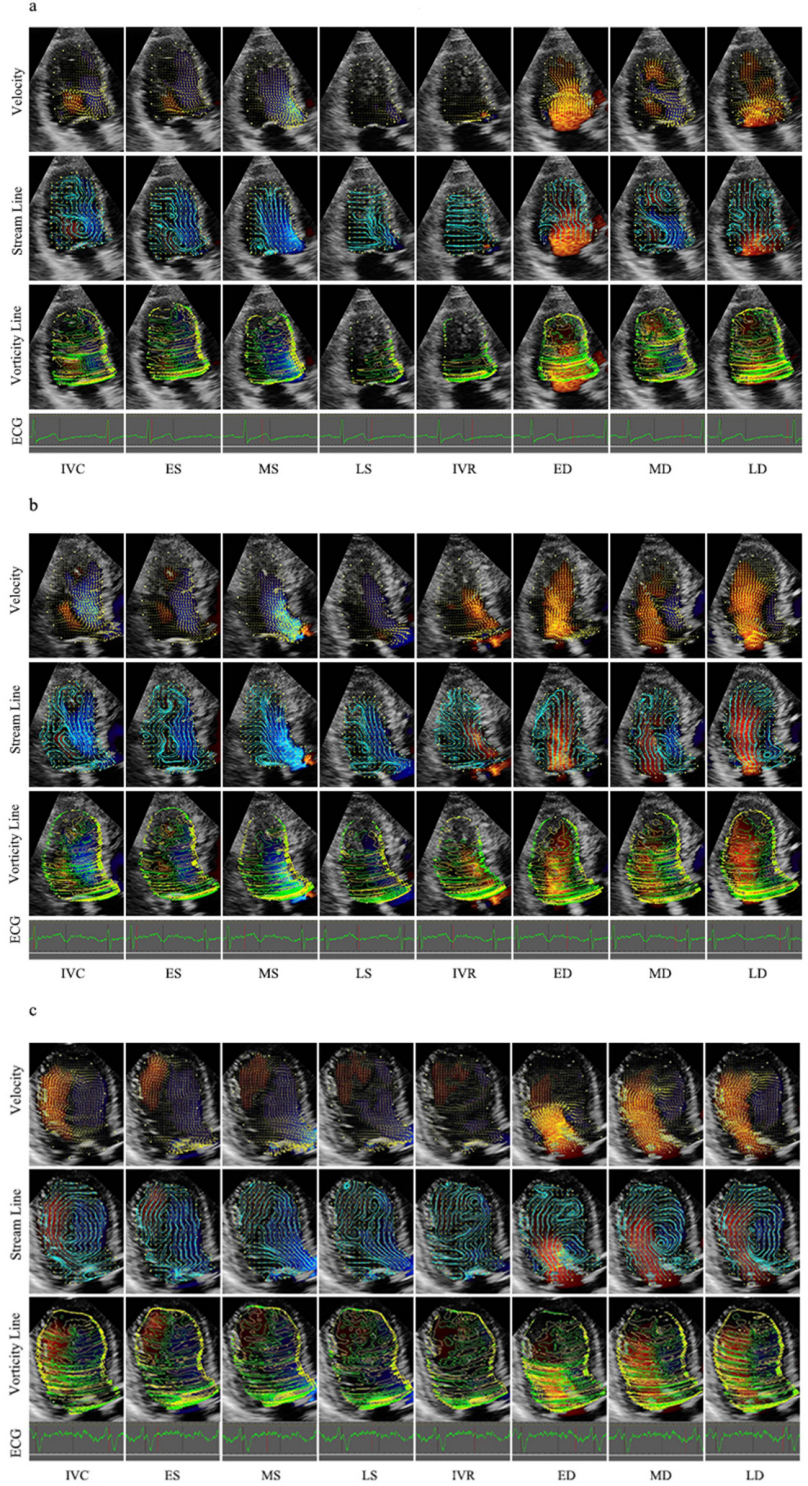
Velocity, stream line and vorticity line. **(a)** Control group; **(b)** Group I; **(c)** Group II. IVR, isovolumic relaxation; ED, rapid filling (early diastole); MD, slow filling (mid-diastole); LD, atrial contraction (late diastole); IVC, isovolumic contraction; ES, early systole; MS, mid-systole; LS, late systole.

In the vector map, the direction is indicated by the inclination and the yellow arrow, whereas the speed is indicated by the length of the yellow line. In the stream line map, a curved line is continuously displayed along the direction of the velocity vector and enables visualization of the vortex rotation. In the vorticity line map, the vorticity is indicated as contour lines and divided into positive and negative areas, which are color-coded. The positive area (counterclockwise direction) is indicated by yellow, and the negative area (clockwise direction) is indicated by green. The maps of these three forms can be seen in Figure 2.

Heart rate was calculated on the basis of the RR interval of the cardiac cycle. On the basis of the synchronous ECG and valve opening‒closing, the cycle was divided into diastole and systole. Diastole was defined as the period from the onset of aortic valve closure to mitral valve closure and was divided into four phases: isovolumic relaxation (IVR), rapid filling (early diastole, ED), slow filling (mid-diastole, MD) and atrial contraction (late diastole, LD). Systole was defined as the remaining part of the cycle and was divided into three phases: isovolumic contraction (IVC), early systole (ES), mid-systole (MS), and late systole (LS). IVR starts from the closure of the aortic valve to the opening of the mitral valve. ED starts from the opening of the mitral valve to the peak of the curve. The MD starts from the peak to the opening of the mitral valve. The LD starts from the opening of the mitral valve to its closure. IVC starts from the closure of the mitral valve to the opening of the aortic valve. ES, which can be rapid ejection, starts from the opening of the aoric valve to the minimum peak of the time‒flow curve. MS and LS, which are slow ejection methods, start from the minimum peak of the time-flow curve to the closure of the aortic valve. The frame at the end of the T wave on a synchronous electrocardiogram was determined as the end of systole (Figure 2).

In this study, Vort-max was measured in each of the eight defined cardiac phases (IVC, ES, MS, LS, IVR, ED, MD, and LD). Through the long axis, from the apex to the middle of the mitral valve annulus, the left ventricle was equally divided into base, middle, and apex segments. The corresponding Vort-max values were defined as Vort-max-base, Vort-max-middle and Vort-max-apex.

### 2.4 Reproducibility

Intra- and interobserver variabilities in Vort-max were analyzed repeatedly in 10 randomly selected subjects. To evaluate intraobserver variability, one observer evaluated the same studies on two separate occasions. To evaluate interobserver variability, two independent observers performed the measurements independently. The variability values were analyzed via Bland‒Altman bias plots.

### 2.5 Statistical analysis

All the statistical analyses were performed with SPSS version 17.0 software (SPSS, Inc., Chicago, IL, USA). Categorial variables were analyzed via the *X*^2^ test. Continuous variables are tested for a normal distribution via the Kolmogorov‒Smirnov test. Normally distributed data are presented as the means ± Standard Error of the Mean (SEM). Skewed distributions are expressed as medians (interquartile ranges). The data were subsequently compared via analysis of equal variance, as they were normally distributed. Echocardiographic parameters were compared by one-way ANOVA. When analysis revealed a significant difference, a Bonferroni *post hoc* comparison test was performed. Correlations between Vort-max and conventional echocardiographic parameters (*E*, *A*, *E*/*A*, EDT, etc.) were evaluated via Pearson’s correlation coefficient. Stepwise multiple regression analyses were used to study the independent factors correlated with Vort-max. Reproducibility was assessed via Bland‒Altman analysis. The analysis was conducted on measurements of the apical segment in ED, MD and LD from ten randomly selected samples. Differences were considered statistically significant at *P* < 0.05.

## 3 Results

### 3.1 General

The descriptive statistics of the clinical characteristics of the study population are summarized in Table 1. However, there were no significant differences among the three groups in terms of age, height, weight, body mass index, or heart rate.

**Table 1:** Clinical characteristics and conventional echocardiographic parameters $\left(\bar{x}\pm \mathrm{SEM}\right)$.

Variables	Controls (*n* = 26)	Group I (*n* = 17)	Group II (IIa/IIb) (*n* = 21)	*P* value
*N*	22	17	21 (7/14)	
Grade I	—	10	14	—
Grade II	—	6	4	—
Grade III	—	1	3	—
Sex (male/female)	15/7	9/8	14/7	—
Age (years)	56.77 ± 2.05	60.06 ± 3.06	60.45 ± 2.93	0.168
Height (cm)	166.40 ± 1.61	165.80 ± 1.64	165.15 ± 1.27	0.959
Weight (kg)	67.33 ± 2.49	66.88 ± 3.18	64.45 ± 2.60	0.467
BSA (m^2^)	1.76 ± 0.03	1.74 ± 0.04	1.71 ± 0.03	0.491
Heart rate (beats/min)	69.35 ± 2.83	68.00 ± 2.97	78.05 ± 4.08	0.081
IVST (mm)	10.70 ± 1.02	12.43 ± 1.38	10.18 ± 1.05	0.388
LVID (mm)	45.64 ± 2.11	45.86 ± 3.58	55.18 ± 4.20	0.163
PWT (mm)	7.82 ± 0.36	8.86 ± 0.80	10.27 ± 0.73	0.100
LVMI (g/m^2^)	80.85 ± 14.07	103.5 ± 17.24	126.7 ± 12.05	0.122
RWT	0.34 ± 0.02	0.39 ± 0.04	0.43 ± 0.08	0.724
LVEDV (ml)	77.09 ± 4.73	91.18 ± 7.83	129.71 ± 10.38*^,^#	<0.001
LVESV (ml)	29.14 ± 2.72	33.00 ± 3.78	90.65 ± 9.27*^,^#	<0.001
SV (mm)	47.95 ± 2.65	58.18 ± 5.19	39.06 ± 3.30^,^#	0.003
*D* (mm)	69.21 ± 1.76	72.30 ± 2.30	75.11 ± 1.82	0.090
*d* (mm)	35.16 ± 1.10	39.17 ± 1.67	48.56 ± 1.38*	<0.001
Sphericity index	0.51 ± 0.01	0.54 ± 0.12	0.65 ± 0.03	0.339
Mitral inflow *E* velocity (cm/s)	75.60 ± 2.96	89.42 ± 10.32	73.44 ± 6.67	0.229
Mitral inflow *A* velocity (cm/s)	59.62 ± 2.75	83.00 ± 4.39*	70.56 ± 4.76	<0.001
*E*/*A* ratio	1.30 ± 0.05	1.18 ± 0.23	1.22 ± 0.21	0.885
EDT (ms)	176.40 ± 9.48	189.60 ± 21.90	126.29 ± 11.26	0.006
*e*′ (cm/s)	12.91 ± 0.62	5.54 ± 0.19*	8.38 ± 0.92*^,^#	<0.001
*E*/*e*′ ratio	5.97 ± 0.25	16.11 ± 1.52*	10.12 ± 1.13*^,^#	<0.001
*E*/*Vp*	1.96 ± 0.20	2.46 ± 0.21	2.52 ± 0.14	0.484
LVEF (%)	64.18 ± 1.36	62.22 ± 1.76	33.07 ± 2.56*^,^#	<0.001

Grade I, impaired relaxation pattern; Grade II, pseudonormal pattern; Grade III, restrictive filling pattern; IVST: Interventricular septal thickness; LVID, left ventricular internal dimension; PWT, posterior wall thickness; LVMI, left ventricular mass index; RWT, relative wall thickness; RWT = 2*PWT/LVID; LVEDV, left ventricular end-diastolic volume; LVESV, left ventricular end-systolic volume; SV, stroke volume; *D*, LV end-diastolic long diameter; *d*, LV end-diastolic short diameter; *E*/*A*, the ratio between mitral inflow *E* velocity and *A* velocity; EDT, *E*-wave deceleration time; *e*′, early diastolic mitral lateral annular velocity; *E*/*Vp*, the ratio between mitral inflow *E* velocity and flow propagation velocity (*Vp*); LVEF, left ventricular ejection fraction.

* *P* < 0.05 vs. controls. #*P* < 0.05 vs. Group I.

### 3.2 Disease distribution in group I and group Ii

In Group I, the most frequent comorbidities were hypertension (6/17) and ischemic heart disease (7/17), followed by obesity (3/17), valvular disease (2/17), diabetes mellitus (1/17), arrhythmia (1/17), and hypertrophic cardiomyopathy (1/17). Group II displayed greater etiological heterogeneity. The main comorbidities included ischemic heart disease (7/21), obesity (4/21), diabetes (3/21), hypertension (2/21), arrhythmia (2/21), valvular disease (1/21), hypertrophic cardiomyopathy (1/21), alcoholic cardiomyopathy (1/21), and dilated cardiomyopathy (2/21) (Supplementary Table 1).

### 3.3 Conventional echocardiography: ventricular geometry

The conventional echocardiographic parameters are shown in Table 1. The LV end-diastolic volume (LVEDV) and LV end-systolic volume (LVESV) were greater in Group II than in the other two groups. LV end-diastolic short diameter (*d*) were greater in Group II than in the other two groups. Other parameters revealed no statistically significant differences in the three groups.

### 3.4 Conventional echocardiography: left ventricle hematography

The peak early mitral diastolic flow (*E*) did not differ significantly. LVEF were lower in Group II than in the other two groups (*P* < 0.001). The peak late mitral diastolic flow (*A*) in Group I were higher than in the other two groups (*P* < 0.001). Early diastolic mitral annular velocity (*e*′) were increased from Group I, Group II to Control Group (*P* < 0.001). *E*/*e*′ were decreased from Group I, Group II to Control Group. (*P* < 0.001). *E*/*Vp* was lower in the control group than in the other groups (*P* < 0.01) (Table 1).

### 3.5 Velocity vector map and streamline map

The left ventricular velocity vector map and vorticity map at representative stages of the whole cardiac cycle are shown in Figure 2 and Table 2, which were obtained from a control patient, a patient with diastolic dysfunction, and a patient with systolic and diastolic dysfunction.

**Table 2:** The values of Vort-max (s^−1^) $\left(\bar{x}\pm SEM\right)$.

Variables	Segment	Control group (*n* = 22)	Group I (*n* = 17)	Group II (*n* = 21)	*P* Value
IVC	Apex	78.44 ± 7.928	94.75 ± 14.89	90.84 ± 49.12	0.558
	Middle	134.60 ± 13.45	167.92 ± 17.54	129.33 ± 15.72	0.227
	Base	209.20 ± 17.83	214.48 ± 26.72	184.40 ± 12.01	0.498
ES	Apex	59.03 ± 4.81	81.41 ± 13.31	83.83 ± 12.31	0.122
	Middle	129.00 ± 15.36	125.35 ± 12.33	108.74 ± 6.87	0.492
	Base	200.70 ± 31.35	219.03 ± 45.20	155.80 ± 15.02	0.376
MS	Apex	44.21 ± 7.97	77.87 ± 11.37*	73.89 ± 8.54	0.017
	Middle	92.87 ± 13.24	104.75 ± 15.07	83.53 ± 5.81	0.526
	Base	217.00 ± 41.77	190.30 ± 34.65	144.11 ± 16.20	0.311
LS	Apex	44.54 ± 6.21	58.35 ± 10.49	61.90 ± 6.56	0.188
	Middle	70.35 ± 5.58	74.36 ± 11.85	77.21 ± 6.81	0.791
	Base	119.00 ± 12.33	135.82 ± 20.36	130.43 ± 25.56	0.818
IVR	Apex	53.75 ± 7.60	39.01 ± 8.27	53.75 ± 7.65	0.391
	Middle	72.81 ± 9.85	62.83 ± 12.68	77.31 ± 7.76	0.641
	Base	141.40 ± 24.17	151.70 ± 49.01	133.55 ± 28.38	0.933
ED	Apex	73.79 ± 8.55	86.60 ± 7.63	58.99 ± 10.57	0.163
	Middle	180.10 ± 24.55	236.22 ± 36.56	161.2 ± 24.52	0.206
	Base	225.01 ± 26.40	351.88 ± 56.23	207.89 ± 41.36	0.051
MD	Apex	106.00 ± 9.20	152.52 ± 51.22	76.72 ± 13.46	0.128
	Middle	137.40 ± 11.04	166.28 ± 21.31	136.13 ± 21.20	0.450
	Base	134.60 ± 15.26	181.00 ± 39.72	163.81 ± 26.35	0.427
LD	Apex	69.38 ± 5.29	95.77 ± 20.17	77.19 ± 14.45	0.361
	Middle	119.30 ± 11.00	206.99 ± 46.17	137.00 ± 23.55	0.062
	Base	204.00 ± 16.17	329.55 ± 35.12*^,^#	251.85 ± 19.06	0.001

IVC, isovolumic contraction; ES, early systole; MS, mid-systole; LS, late systole; IVR, isovolumic relaxation; ED, rapid filling (early diastole); MD, slow filling (mid-diastole); LD, atrial contraction (late diastole).

* *P* < 0.05 vs. controls. #*P* < 0.05 vs. Group I.

During isovolumic contraction, a large vortex was generated along the septum, which was more irregular in Group I and Group II. In systole, the flow accelerated from the apex toward the outflow tract, and then, a part of the flow turned from the outflow to the anterior mitral leaflet. Hence, the oblong vortex was generated in the upper middle of the LV and dissipated quickly. However, flow toward the apex was winding along the posterior wall in Group I, whereas some tiny vortexes remained at the apex and persisted longer in Group II. The early diastolic flow spreads toward the apex, with small vortices curling back beneath the anterior and posterior mitral leaflets. In mid-diastole, a clockwise round or oblong vortex arose in the whole cavity or at the middle segment. However, the flow still dominantly spread toward the apex in part of Group I, and a flow circle occupied the whole LV chamber with several small disordered vortexes and persisted through diastole in most of Group II. At the end of diastole, several small vortexes were beneath the anterior and posterior mitral leaflets, with the anterior ones being larger, whereas the vector in Group I was irregular in the base segment.

### 3.6 Vorticity line map: Vort-max, vortex circulation and area compared in different segments

**Figure 3:**
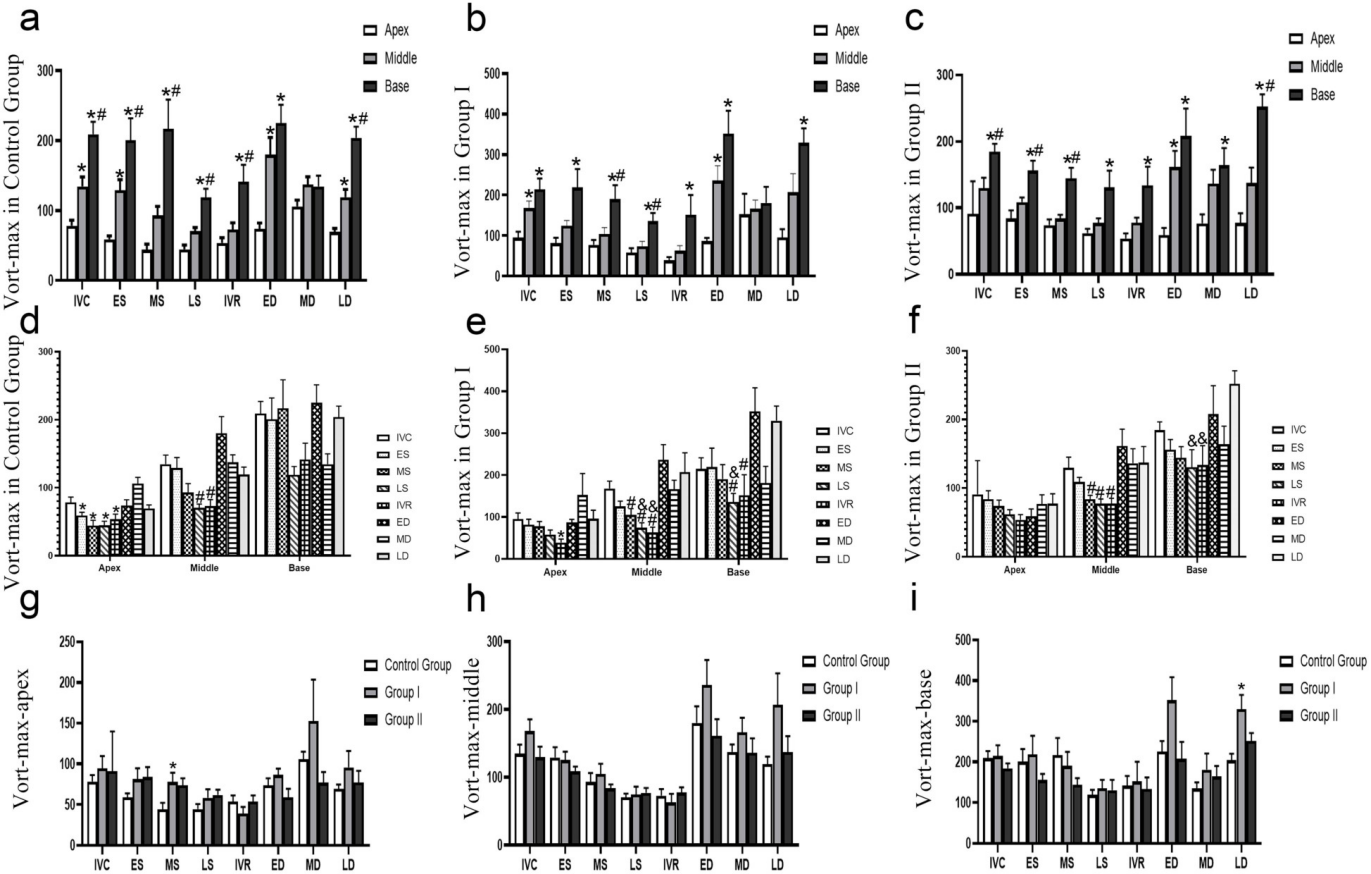
Evolution of vort-max in different groups. **(a–c)** The Vort-max differences in the base, middle and apex segments, *P < 0.05, vs. apex segment, #*P* < 0.05, vs. mid segment, **(d–e)** the Vort-max differences in different times, *P < 0.05, vs. MD, #*P* < 0.05, vs. ED, & *P* < 0.05, vs. LD, **(f–i)** the Vort-max differences in different groups, *P < 0.05, vs. Control Group, #*P* < 0.05, vs. Group I. IVC, isovolumic contraction; ES, early systole; MS, mid-systole; LS, late systole; IVR, isovolumic relaxation; ED, rapid filling (early diastole); MD, slow filling (mid-diastole); LD, atrial contraction (late diastole).

The differences in the base, middle and apex segments are shown in Figures 3a–c. The measured values of Vort-max are shown in Table 2.

#### (1) Control group: a clear base-to-apex gradient

In the control group, the parameter exhibited a significant and dynamic spatial heterogeneity.

During isovolumic contraction (IVC), early ystole (ES), and late diastole (LD), a clear base-to-apex descending gradient was observed, with values progressively and significantly decreasing from the basal to the mid and apical segments (*P* < 0.05).

Conversely, during mid-systole (MS), late systole (LS), and isovolumic relaxation (IVR), the basal segment showed significantly higher values than both the mid and apical segments (*P* < 0.05), which themselves were not significantly different from each other.

At early diastole (ED), the apical segment value was significantly lower than that of the mid and basal segments (*P* < 0.05), with no difference between the latter two.

No significant differences were found among the three segments during mid-diastole (MD).

#### (2) Group I: blunted gradients with preserved basal dominance in systole

Group I demonstrated a partial alteration of the normal spatial pattern. The clear base-to-apex gradient seen in controls was less pronounced. During ES, IVR, and LD, only the difference between the basal and apical segments remained statistically significant (*P* < 0.05), while differences between adjacent segments (base vs. mid, mid vs. apex) were non-significant. At IVC and ED, the pattern mirrored the control group’s ED phase, with the apical segment showing significantly lower values than the non-different basal and mid segments. Notably, the systolic pattern of basal dominance was preserved: during MS and LS, the basal segment value was significantly higher than both the mid and apical segments (*P* < 0.05), which were statistically similar. Similar to the control group, no segmental differences were observed during MD.

#### (3) Group II: a persistent “base-dominant” pattern

Group II exhibited a more profound and consistent alteration in spatial distribution, characterized by a persistent dominance of the basal segment.

Across a majority of the cardiac cycle—specifically during IVC, ES, MS, and LD—the parameter value in the basal segment was significantly higher than in the mid and apical segments (*P* < 0.05), between which there was no statistical difference.

During LS, IVR, and MD, the pattern shifted, showing a significantly lower value in the apical segment compared to the basal segment (*P* < 0.05), but with no significant differences between adjacent segments.

At ED, the apical segment value was significantly lower than both the mid and basal segments (*P* < 0.05), which were similar to each other.

In conclusion, the spatial distribution of this parameter within the left ventricle demonstrates distinct patterns across the three groups, reflecting different underlying physiological and pathological states.

In the control group, a clear spatial heterogeneity was observed, characterized by a significant base-to-apex descending gradient during isovolumic contraction, end-systole, and late diastole. This gradient was replaced by a pattern of basal segment dominance during mid-to-late systole and isovolumic relaxation.

Group I exhibited a partial disruption of this normal pattern. The base-to-apex gradient during contraction and relaxation phases was significantly blunted, indicating a less heterogeneous spatial distribution. However, the systolic dominance of the basal segment was preserved, suggesting a transitional or less severe pathological state.

In stark contrast, Group II showed a fundamental shift in its spatial signature. The physiological gradients were largely replaced by a persistent “base-dominant” pattern across most of the cardiac cycle. This suggests a significant and consistent alteration in intraventricular dynamics.

Overall, these findings indicate that segmental analysis of this parameter can effectively differentiate between the three groups. The progressive change from a clear spatial gradient (Control Group) to a blunted gradient (Group I), and finally to a persistent base-dominant pattern (Group II), may serve as a quantitative marker for characterizing and staging the underlying cardiac condition.

### 3.7 Vorticity line map: Vort-max, vortex circulation and area compared at different times

The differences at different times are shown in Figures 3d–f.

#### 3.7.1 Control group: dynamic apical/mid segments and a stable base

In the control group, significant temporal variations were primarily localized to the apex and middle segments, while the base segment remained stable. Values during ES, MS, LS, and IVR were all significantly lower compared to the value at MD in apex (*P* < 0.05). A significant decrease was observed during LS and IVR when compared to ED in middle (*P* < 0.05). No significant variations were found across any of the analyzed cardiac phases in base.

#### 3.7.2 Group I: emergence of dynamic changes in the basal segment

Group I demonstrated altered temporal dynamics, most notably with the emergence of significant variations in the base segment.

The temporal variation was less pronounced than in controls, with a significant decrease observed only during IVR compared to MD in apex (*P* < 0.05). In middle segment, this segment showed extensive variations. Values during MS, LS, and IVR were all significantly lower than at ED. Furthermore, the value at LS was lower than at LD, and the value at IVR was lower than both ED and LD (*P* < 0.05 for all). In contrast to the stable control base, this segment became dynamic. Values during LS were significantly lower than at both ED and LD, and the value during IVR was significantly lower than at ED in base (*P* < 0.05).

#### 3.7.3 Group II: loss of apical dynamics and persistent Mid/basal changes

Group II exhibited a further shift in temporal patterns, characterized by a loss of apical dynamics.

No significant differences were found across the cardiac cycle in apex, indicating a loss of the temporal variability seen in Group II. The pattern of systolic reduction was maintained, with values during MS, LS, and IVR being significantly lower than at ED in middle (*P* < 0.05).The dynamic changes persisted, with values during LS and IVR being significantly lower compared to LD in base (*P* < 0.05).

In conclusion, this study identifies a clear, progressive alteration in the heart’s temporal dynamics that correlates with disease state. A healthy heart is characterized by dynamic apical/mid-segments and a stable base. Disease progression is marked by a distinct functional reversal: the apical segment becomes static, while the basal segment develops pathological dynamic changes. This shift from apical dynamism to basal instability serves as a potent marker of cardiac pathology.

### 3.8 Vorticity line map: Vort-max, vortex circulation and area compared in different groups

The differences among the three groups are shown in Figures 3g–i. In the base segment, the Vort-max of the LD in Group I was greater than that in Control Groups (*P* < 0.05). In the apex segment, the Vort-max of the LD in Group I was greater than that in the Control Group (*P* < 0.05).

### 3.9 Correlation analysis with LV diastolic function

The observed vortexes were correlated with the velocity of the ventricular filling waves. The strongest associations were found with indices of diastolic dysfunction. Specifically, numerous parameters, particularly those measured in mid-to-late diastole, showed strong positive correlations with *E*/*e*′, a primary indicator of elevated left ventricular filling pressures (Table 3).The studied parameters hold potential as quantitative markers for assessing the severity of diastolic dysfunction.

**Table 3:** The correlations between Vort-max and conventional echocardiographic parameters.

Variables	*T*(*e*′ − *E*)		*E*/*e*′		*E*		*A*	
	*r*	*P*	*r*	*P*	*r*	*P*	*r*	*P*
Apex of IVC	−0.297	0.053	0.275	0.068	0.573**	<0.001	0.424**	0.004
Mid of IVC	−0.256	0.098	0.160	0.292	0.299*	0.046	0.463**	0.001
Base of IVC	0.212	0.173	−0.065	0.673	0.006	0.967	−0.033	0.832
Apex of ES	−0.254	0.101	0.214	0.159	0.458**	0.002	0.304*	0.042
Mid of ES	−0.198	0.202	−0.117	0.444	−0.182	0.232	−0.115	0.450
Base of ES	−0.162	0.300	−0.029	0.852	0.000	0.999	−0.128	0.403
Apex of MS	−0.375*	0.013	0.400**	0.006	0.251	0.096	0.272	0.070
Mid of MS	−0.236	0.128	0.144	0.345	0.016	0.918	−0.004	0.977
Base of MS	−0.142	0.365	0.010	0.946	−0.063	0.681	−0.291	0.053
Apex of LS	−0.107	0.495	0.230	0.128	0.213	0.160	0.142	0.352
Mid of LS	−0.374*	0.013	0.198	0.192	0.193	0.204	0.069	0.651
Base of LS	−0.367*	0.015	0.289	0.054	0.246	0.104	−0.114	0.456
Apex of IVR	−0.187	0.231	−0.122	0.426	0.038	0.805	0.107	0.484
Mid of IVR	−0.146	0.352	−0.118	0.429	0.111	0.467	0.059	0.699
Base of IVR	−0.062	0.691	−0.115	0.452	−0.017	0.909	−0.045	0.770
Apex of ED	−0.285	0.064	−0.027	0.859	−0.032	0.836	−0.026	0.864
Mid of ED	−0.260	0.093	0.322*	0.031	0.413**	0.005	0.065	0.670
Base of ED	−0.317*	0.039	0.507**	<0.001	0.628**	<0.001	0.175	0.250
Apex of MD	−0.629**	<0.001	0.395**	0.007	0.540**	<0.001	0.289	0.054
Mid of MD	−0.511**	<0.001	0.397**	0.007	0.686**	<0.001	0.270	0.073
Base of MD	−0.371*	0.014	0.479**	<0.001	0.496**	<0.001	0.270	0.073
Apex of LD	−0.503**	<0.001	0.303*	0.043	0.514**	<0.001	0.153	0.316
Mid of LD	−0.364*	0.016	0.416**	0.005	0.369*	0.013	0.340*	0.022
Base of LD	−0.082	0.603	0.444**	0.002	0.121	0.427	0.492**	<0.001

IVC, isovolumic contraction; ED, rapid filling (early diastole); MD, slow filling (mid-diastole); LD, atrial contraction (late diastole).

* *P* < 0.05. **P < 0.01.

A significant positive correlation with *E*/*e*′ was found for numerous parameters, most notably apex of MS (*r* = 0.400, *P* = 0.006), mid of ED (*r* = 0.322, *P* = 0.031), base of ED (*r* = 0.507, *P* < 0.001), apex of MD (*r* = 0.395, *P* = 0.007), mid of MD (*r* = 0.397, *P* = 0.007), base of MD (*r* = 0.479, *P* < 0.001), apex of LD (*r* = 0.303, *P* = 0.043), mid of LD (*r* = 0.416, *P* = 0.005), and base of LD (r = 0.444, *P* = 0.002).

The E value was strongly and positively correlated with apex of IVC (*r* = 0.573, *P* < 0.001), mid of IVC (*r* = 0.299, *P* = 0.046), apex of ES (*r* = 0.458, *P* = 0.002), and was particularly correlated with parameters in mid-to-late diastole such as base of ED (*r* = 0.628, *P* < 0.001), apex of MD (*r* = 0.540, *P* < 0.001), mid of MD (*r* = 0.686, *P* < 0.001), base of MD (*r* = 0.496, *P* < 0.001), and apex of LD (*r* = 0.514, *P* < 0.001). The A value was also positively correlated with apex and mid of IVC, apex of ES, mid and base of LD.

The *T*(*e*′ − *E*) showed a consistent and strong negative correlation with diastolic phase parameters, including base of ED (*r* = −0.317, *P* = 0.039), apex of MD (*r* = −0.629, *P* < 0.001), mid of MD (*r* = −0.511, *P* < 0.001), base of MD (*r* = −0.371, *P* = 0.014), apex of LD (*r* = −0.503, *P* < 0.001), and mid of LD (*r* = −0.364, *P* = 0.016). Furthermore, apex of MS also had a significant negative correlation with *T*(*e*′ − *E*) (*r* = −0.375, *P* = 0.013). The consistent negative correlation with *T*(*e*′ − *E*) further reinforces the association with impaired myocardial relaxation.

### 3.10 Intra- and interobserver variability

Bland‒Altman analysis demonstrated that Vort-max measurements from different segments exhibited good reproducibility (Supplementary Figure 1).

The intra- and interobserver reproducibility for Vort-max measurements was excellent. Bland–Altman analysis for intraobserver variability of Vort-max in apex demonstrated a mean bias of 0.125 s^−1^ with 95% limits of agreement of −1.293 to 1.543 s^−1^ in ED, 0.221 s^−1^ with 95% limits of agreement of −2.078 to 2.520 s^−1^ in MD, and 0.056 s^−1^ with 95% limits of agreement of −0.860 to 1.543 s^−1^ in LD. For interobserver variability of Vort-max in apex, the mean bias was −1.123 s^−1^ with 95% limits of agreement of −5.326 to 2.081 s^−1^ in ED,−2.484 s^−1^ with 95% limits of agreement of −6.637 to 1.668 s^−1^ in MD, and −1.616 s^−1^ with 95% limits of agreement of −3.948 to 0.717 s^−1^ in LD.

## 4 Discussion

The findings of this study demonstrate that echocardiography-based VFM provides a quantitative and feasible bedside assessment of intraventricular hemodynamics. Abnormal hemodynamics may accompany cardiac dysfunction and may be one of the potential causes of these diseases (20, 29–31). Our results show that Vort-max, a key parameter of vortex dynamics, offers novel insights into the pathophysiology of cardiac dysfunction, allowing for the differentiation of normal hearts from those with either isolated diastolic or combined systolic-diastolic failure.

In our cohort of 60 subjects, we identified distinct intraventricular vorticity signatures for each functional group. Healthy hearts displayed regular, synchronized flow patterns consistent with efficient energy transfer through the cardiac cycle. In contrast, patients with isolated diastolic dysfunction (Group I) exhibited a significantly elevated Vort-max during early diastole, reflecting impaired relaxation and augmented inflow forces. Furthermore, patients with combined systolic and diastolic dysfunction (Group II) showed abnormal vortex persistence at the LV apex and reduced systolic Vort-max, signifying inefficient contraction, energy trapping, and impaired ejection.

These findings align with the concept that diastolic dysfunction often represents the earliest and most sensitive pathophysiologic change in cardiac disease (21, 32). Intraventricular vortices are fundamental hemodynamic structures that optimize blood transport by preserving kinetic energy and minimizing energy dissipation (1, 10). Pathological cardiac remodeling—including hypertrophy, fibrosis, or dilatation—disrupts this delicate balance, leading to disorganized vortical flow and inefficient pump function (19). Our findings provide a direct hemodynamic link to this process.

In diastolic dysfunction, increased myocardial stiffness impairs ventricular relaxation. This forces a more rapid and turbulent early diastolic filling to maintain cardiac output, providing a direct mechanistic explanation for our observation of an elevated early-diastolic Vort-max. Conversely, the persistent apical vortices and weaker systolic Vort-max in patients with systolic failure demonstrate profound energetic inefficiency. The dilated, poorly contracting ventricle is unable to effectively form a functional vortex or redirect blood toward the outflow tract, causing energy to be trapped and dissipated within the LV cavity (33).

These hemodynamic signatures align with the distinct etiologies of our patient groups. Group I was primarily characterized by hypertension and concentric hypertrophy, classical mechanisms that cause restrictive filling patterns and thus generate a high-energy diastolic vortex. Group II had a higher prevalence of ischemic and dilated cardiomyopathies, which lead to ventricular dilation and loss of contractile efficiency. This pathophysiology explains the weaker, disorganized vortex patterns observed, reflecting a global failure of both energy generation (systole) and efficient energy transfer (diastole).

While pioneering work using 4D Flow MRI established the link between altered LV vorticity and cardiac disease, its clinical application remains limited by high cost, complexity, and lack of routine accessibility (3, 4). Our study confirms that echocardiography-based VFM can detect these critical alterations in vortex dynamics at the bedside, offering a significant practical advantage for clinical research and potential future practice. Although it has been reported that vorticity changes in patients with heart failure are different from those in control individuals, the degree of the abnormal velocity vector field still unclear (34–36).

Previous echocardiographic studies have often focused on qualitative or morphological descriptions of vortices (e.g., vortex length, width, or persistence) to assess cardiac function (5, 12–14, 18–20). Our work advances this field by using Vort-max to provide a direct, angle-independent quantification of rotational blood flow. Unlike traditional Doppler indices, which are angle-dependent and measure velocity in a single direction, Vort-max integrates both the magnitude and direction of velocity vectors across a 2D plane, offering a more comprehensive and potentially more robust metric of intraventricular hemodynamics (28).

The quantitative nature of Vort-max suggests several potential clinical applications. It may aid in the diagnosis and stratification of diastolic dysfunction (10, 13, 14, 20, 33, 34, 37, 38), particularly in cases where conventional indices are indeterminate, by providing objective evidence of inefficient intraventricular flow.

A common phenotype of diastolic dysfunction, particularly in patients with hypertension, involves compensatory left ventricular hypertrophy and a resultant hyperdynamic contractile state. We propose that during isovolumic contraction, the vigorous contraction of these hypertrophied basal segments rapidly accelerates intraventricular blood, leading to the formation of a more intense vortex. This provides a direct mechanistic explanation for our finding of significantly elevated Vort-max vorticity in the basal segment. Diastolic dysfunction can also occur due to other factors such as aging, diabetes mellitus, coronary artery disease, and various cardiomyopathies (39).

Looking forward, Vort-max could serve as a novel prognostic marker. It is plausible that disorganized flow patterns, as quantified by Vort-max, may predict adverse clinical outcomes independent of LVEF. Furthermore, Vort-max could be explored as a novel endpoint to assess the hemodynamic efficacy of therapies such as cardiac resynchronization or new pharmacological agents (40).

This study has several limitations. The most significant is the relatively small sample size, which precluded robust statistical comparisons between all HF subgroups and limited our ability to correlate findings with long-term clinical outcomes. Second, VFM requires adequate acoustic windows and may not capture the entire LV in severely dilated hearts. Thirdly, the VFM algorithm is based on the assumption of a laminar, incompressible Newtonian fluid. In severely diseased states with high-velocity jets or significant turbulence, this assumption may be violated, potentially affecting the accuracy of the velocity vector calculations. However, VFM is designed to capture the large, organized vortical structures rather than micro-turbulent flow. Finally, this investigation did not include a direct comparison to a gold-standard reference, such as catheter-based pressure measurements or 4D Flow MRI. Therefore, larger-scale, prospective studies are warranted to validate these phenotype-specific Vort-max profiles and to establish the prognostic value of this metric across the full spectrum of heart failure.

## Data Availability

The raw data supporting the conclusions of this article will be made available by the authors, without undue reservation.
