# Supplementary material for: Evaluation of the intraventricular hemodynamics of patients with left ventricular dysfunction via vector flow mapping
Source: Front Cardiovasc Med. 2025 Oct 15;12:1617482. doi: 10.3389/fcvm.2025.1617482 (PMC12568562; doi:10.3389/fcvm.2025.1617482)
Supplement: Supplementary file 1 [file Table1.docx]

**[Supplement](http://www.baidu.com/link?url=uVMVE79NFa6c4L6MF6FyOq3c5w4aPpU4Ccp3K5hzsmzIAPXq4DlMIUIlRBzczERgO5ztRsIhTHGljj6jPRFyB_) Table 1：Disease distribution in Group I and Group II**

|  | Group I(n=17) | Group II(n=21) |
| --- | --- | --- |
| Hypertension | 6 | 2 |
| Ischemic heart disease | 7 | 7 |
| Arrhythmia | 1 | 2 |
| Valvular heart disease | 2 | 1 |
| Diabetes mellitus | 1 | 3 |
| Obesity | 3 | 4 |
| Hypertrophic cardiomyopathy | 1 | 1 |
| Alcoholic cardiomyopathy | 0 | 1 |
| Dilated cardiomyopathy | 0 | 2 |
| Pulmonary arterial hypertension | 0 | 2 |
